# To the Editor—What should we expect from Omnipolar mapping technology during cardioneuroablation?

**DOI:** 10.1016/j.hrcr.2024.09.025

**Published:** 2024-10-12

**Authors:** Tolga Aksu

**Affiliations:** Yeditepe University Faculty of Medicine, İstanbul, Turkey

I read with great interest the article “Cardioneuroablation Guided by Real-Time Spectral Analysis: The Omnipolar Technology Near Field” by Giomi and colleagues.^1^ In the article, the authors compared a fractionation mapping tool with the Omnipolar Technology Near Field of the EnSite system (Abbott) for ganglionated plexus (GP) mapping. A mismatch between frequency and fragmentation maps was observed in the superior right atrial GP and superior left atrial GP, where high-frequency potentials did not show equally high fragmentation.

A fractionation mapping tool was first used to map GP sites by our group.^2^ By using ablation or high-density mapping catheters, the system allowed us to calculate the number of electrogram deflections and encode fractionation areas on the basis of the defined fractionation threshold.^2^^,^^3^ Theoretically, GP sites are characterized by highly fractionated atrial electrograms due to endocardial and myocardial nerve inputs of GP, contrary to the surrounding atrial myocardium with nonfractionated or less fractionated atrial electrograms.

We recently used fractionation mapping software to map the fractionated areas and compared these areas with peak frequency mapping in order to determine whether the EnSite Omnipolar Technology Near Field algorithm could be implemented to reduce the size of the ablation area.^4^

The peak frequency detection in the near field was configured at 400 Hz, 500 Hz, and 600 Hz. The intersection areas of regions with a fractionation mapping score >2 and regions with a peak frequency value >600 Hz were selected as the target for the GP. In case of discrepancy between 2 maps in the indicated regions, areas with a peak frequency value >600 Hz were targeted. In our experience, fractionation mapping and peak frequency mapping demonstrated a good correlation in all GP areas except the inferior left atrial GP, which was not detected by fractionation mapping. However, peak frequency mapping with a cutoff of 600 Hz was limited the target area in each GP, which was compatible with the results of the case report.

A combination of fractionation and peak frequency mapping tools may help less experienced electrophysiologists to concentrate more careful mapping to localize GPs at the areas detected by auto-algorithm. As mentioned in a recently published scientific statement on cardioneuroablation,^5^ comparative data for different GP mapping techniques are lacking and validation studies are needed.
